# Effect of Different Ratios of Gasoline-Ethanol Blend Fuels on Combustion Enhancement and Emission Reduction in Electronic Fuel Injection Engine

**DOI:** 10.3390/polym15193932

**Published:** 2023-09-29

**Authors:** Yanshuai Ye, Jingyi Hu, Zhiqing Zhang, Weihuang Zhong, Ziheng Zhao, Jian Zhang

**Affiliations:** 1 Liuzhou Key Laboratory of Automobile Exhaust Control Technology, Guangxi University of Science and Technology, Liuzhou 545006, China; yeyanshuai@gxust.edu.cn; 2 School of Mechanical and Automotive Engineering, Guangxi University of Science and Technology, Liuzhou 545006, China; 221076847@stdmail.gxust.edu.cn (W.Z.); 221076846@stdmail.gxust.edu.cn (Z.Z.); 20220103103@stdmail.gxust.edu.cn (J.Z.)

**Keywords:** multi-objective optimization, combustion characteristics, emission characteristics, gasoline-cassava bioethanol fuel blends

## Abstract

The severity of engine emissions for the environment and human health cannot be ignored. This article optimizes the combustion and emission of gasoline-cassava bioethanol fuel blends in electronic fuel injection engines using response surface methodology to achieve the goal of reducing carbon and pollutant emissions. The experiment investigated the effects of different gasoline-cassava bioethanol mixing ratios (G100, G90E10, G80E20, and G70E30) on engine performance, including torque, brake specific fuel consumption, power, total hydrocarbons, nitrogen oxides, and carbon monoxide emissions. The results show that the gasoline-cassava bioethanol fuel blend is not as good as G100 in terms of braking power, torque, and brake specific fuel consumption, but better than G100 in terms of carbon monoxide emissions and total hydrocarbon emissions. Then, the optimization objective function was determined, and the combustion and emission characteristics were optimized using the response surface methodology method. The optimization results indicate that the response surface methodology method can determine the interaction between design variables such as brake specific fuel consumption, nitrogen oxides, and total hydrocarbon emissions and find the best solution. In this experiment, the independent variables of the best solution were 72.9 N·m torque, 30% G70E30 mixing rate, and 2000 rpm speed, corresponding to brake specific fuel consumption at 313 g/(kW·h), nitrogen oxide emissions at 2.85 × 10^3^ ppm, and total hydrocarbon emissions at 166 ppm. The findings of this study indicate that by optimizing the gasoline-cassava bioethanol mixture ratio, lower emission levels can be achieved in electronic fuel injection engines, thereby promoting the sustainable development of renewable energy and reducing pollutant emissions.

## 1. Introduction

Despite the rapid development of electric vehicles, traditional fuel vehicles still dominate at present [1]. At the end of March 2022, there were 307 million fuel-fired vehicles, accounting for 76.3% of China’s motor vehicle fleet [2]. Gasoline is mainly used in carbureted gasoline engines, which have the advantages of high efficiency, well smoothness, batter combustion characteristics, and working stability [3,4]. However, if conventional fuels are used alone, this can lead to a scarcity of conventional fuels. The shortage of petroleum and strict emission requirements have promoted the development of alternative fuels for internal combustion engines while also placing higher demands on the technological progress of internal combustion engines [5,6]. Pollutant emissions from vehicles are a major contributor to environmental pollution [7,8]. Vehicle emissions mainly include total hydrocarbons (THC), carbon monoxide (CO), nitrogen oxides (NO_x_), and so on [9]. Exhaust emissions not only cause environmental pollution but also have irreversible effects on human health (e.g., respiratory system, heart disease, diabetes) [10].

Ethanol, as a biofuel, has many desirable physical and chemical properties that make it suitable for use in petrol engines and can improve a large number of engine performances [11,12]. The production of ethanol consists mainly of polymer conversion and chemical synthesis [13,14]. The chemical synthesis method produces toxic substances during the production process and is not suitable for widespread use [15,16]. Polymer-produced ethanol is a promising development for current renewable energy sources as well as for traditional alternative fuels. Ethanol can be produced by the cleavage of a variety of polymers, the most common of which are biomass materials that are high in cellulose and starch (e.g., in cassava and cereal) [17,18]. These polymers can be chemically or biologically cleaved to produce ethanol [19,20]. Polymer production of ethanol has better sustainability than conventional ethanol production methods [21]. It utilizes biomass resources such as waste crops, forest waste, or energy crops, reducing dependence on finite fossil fuels. In addition, the process of polymer production of ethanol produces low greenhouse gas emissions, which can have a positive impact on reducing climate change by significantly reducing the release of carbon dioxide relative to traditional petroleum-based fuels [22]. Ethanol has good compatibility with petrol [23]. Research and practical results have shown that it is feasible to burn gasoline-cassava bioethanol fuel blends in gasoline engines, which reduces the emissions of THC, CO, and NO_x_ while maintaining the engine’s power performance [24,25].

Because of the phase separation of oil and water, the main fuel ethanol used today is anhydrous ethanol with an ethanol content of not less than 99.2% (*v*/*v*) [26,27]. Compared to pure ethanol, gasoline-cassava bioethanol fuel blends have a significant price advantage and low water resistance [28]. By using appropriate emulsifiers, aqueous ethanol with an ethanol content of 95% (*v*/*v*) can also be mixed with gasoline, making gasoline-cassava bioethanol fuel blend mixtures a viable fuel. Brazil was the earliest country to use fuel ethanol. In 2015, the Brazilian government set the volume fraction of gasoline mixed with anhydrous ethanol at 27.0% [29]. Compared to pure gasoline, the use of gasoline-cassava bioethanol fuel blends not only vitally reduces the cost of use, but also has the effect of reducing pollutant emissions while maintaining the same engine dynamics [30].

In recent years, many researchers have investigated gasoline-cassava bioethanol fuel blends [31]. In spark ignition (SI) engines, the blended fuel of G80E20 accelerated the deterioration of engine emissions and performance, as provided by Akansu et al. [32]. For the same SI engine in the study, Thangavel et al. [33] showed that G50E50 had better thermal efficiency and torque only with the fuel mixture. Mohammed et al. [34] also concluded that the higher the ethanol content in SI engines, the better the brake power, brake specific fuel consumption (BSFC), and thermal efficiency. Rao et al. [35] considered that the mixture of G70E30 was the best and had better combustion efficiency. And they found that the mixture of G60E40 was better for combustion at low speed, while pure gasoline without ethanol addition (G100) was better at high speed. Bielaczyc et al. [36], Quiroga et al. [37], and others concluded that the exhaust emissions of ethanol blends were lower than those of gasoline. Ismail et al. [38] found in a study on gasoline-cassava bioethanol fuel blends that speed also had an effect on the experimental results. The increase in speed resulted in a reduction in exhaust emissions. Cordeiro de Melo et al. [39] found that the addition of hydrogenated ethanol reduced CO and THC emissions, but increased CO_2_ and NO_x_ emissions and showed a complex trend with ethanol addition, with results depending on operating conditions, spark advance, and other parameters. In a recent study, Li et al. [40] prepared five kinds of ethanol with different ω (0, 5%, 10%, 15%, and 20% vol.) to research the effects of ethanol on combustion and emissions. The results showed gasoline-cassava bioethanol fuel blends prolonged flame development and propagation duration; CO decreased by 41.9%, 28.5%, 5.59%, and 2.46%; and NO_x_ decreased by 10.1%, −0.75%, −0.17%, and 4.18%. Response surface methodology (RSM) is a simple and effective optimization method that can achieve the goal of optimizing target variables by combining decision variables. The RSM method has been widely applied to optimize engine performance and emissions [41]. Najafi et al. [42] used RSM to optimize gasoline-cassava bioethanol fuel blends using E5, E7.5, E10, E12.5, and E15 on SI engines. The results indicate that using a mixture of G90E10 at a speed of 3000 rpm is the optimal value. However, in their study, a higher ethanol blending ratio was not used. This leads to certain limitations in the data. Yu et al. [43] have conducted energy assessments on cassava fuel ethanol in the Guangxi region in terms of total energy coefficient, net energy value, and energy consumption. The results indicate that cars using G90E10 can save an average of 3.66% in energy. However, the article only measured the mixing ratio of G90E10 and did not conduct experiments on higher proportions of gasoline-ethanol fuel blends. Ethanol-gasoline blended fuel has been widely adopted worldwide. This mixed fuel is usually represented as E10, which contains 10% ethanol and 90% gasoline. In some countries, other mixing ratios can also be found, such as E85 (85% ethanol and 15% gasoline) [44]. Ten percent gasoline-ethanol blend fuel has been recognized by most countries. Ethanol is also being studied for blending with diesel fuel. Augustine et al. [45] used neural network algorithms to optimize brake thermal efficiency, CO, HC, and NO_x_. They found that adding 4% to 6% ethanol, 1.5% emulsifier, and a speed of 2000 rpm can maximize brake thermal efficiency and minimize CO, HC, and NO_x_ emissions. In Brazil, the United States, Canada, and other countries, over 10% of ethanol-gasoline blends have been used, and some European countries even use higher proportions of ethanol-blended fuel. So, the ethanol blending ratio obtained by this method has certain drawbacks. Mokhtar et al. [46] studied the practicality of 60% diesel fuel and 40% biodiesel (B40) and a blend of 60% diesel fuel, 30% biodiesel, and 10% hydrogenated vegetable oil (HVO) (B30D10). They demonstrated through actual road testing experiments that B40 and B30D10 are suitable for operation in low ambient temperatures in Indonesia. Increasing the blending ratio of ethanol in gasoline has become a trend. The current research on gasoline-ethanol blended fuel is summarized in Table 1.

This article uses polymers to prepare cassava bioethanol and blends these materials with gasoline to obtain a green and clean fuel. In this experiment, the fuel obtained by mixing cassava bioethanol and gasoline by the polymer conversion method was used to measure the exhaust emission parameters (including NO_x_, CO, and THC), combustion performance parameters (including BSFC and exhaust temperature), braking power, and torque of this biofuel. Then, in the next step of research, the multi-objective optimization method is used to determine the best performance and the lowest emission level of the gasoline engine. The most exciting part of this study is to improve the ethanol blending ratio of gasoline-cassava bioethanol blended fuel currently used and provide guidance for optimizing fuel formulation and engine tuning so as to reduce fuel consumption, improve power performance, and reduce pollutant emissions. In practical applications, similar optimization methods in this article can also be used to help find the most suitable mixing ratio for other types of engines. This approach can improve fuel efficiency, reduce fuel consumption, and reduce vehicle operating costs. In addition, this can provide consumers with more choices. This helps to meet the needs of different regions, models, and uses.

## 2. Materials and Methods

### 2.1. Preparation and Characterization of Test Fuels

Firstly, cassava with a starch content of 72.4% was used as raw materials for two-stage pulping and sand removal pretreatment and then mixed with sweet sorghum juice with a total sugar content of 14.2% to obtain a pH of around 5.5 to prepare powder slurry. Subsequently, 10 U/g high-temperature-resistant amylase was added and quickly heated to 85–90 °C to convert starch into small-molecule dextrin. The liquefied mash was cooled and mixed with yeast and saccharifying enzymes. Under the conditions of fermentation temperatures of 30–35 °C and 60–75 h, mature alcohol mash was prepared through synchronous saccharification and fermentation operations. Mature mash undergoes multi-stage thermally coupled distillation, molecular sieve temperature, and pressure swing adsorption dehydration to remove most of the water and impurities, obtaining fuel ethanol products. The waste mash is sent to the wastewater treatment unit for biochemical treatment. After undergoing primary anaerobic treatment at 55–65 °C, biogas is collected, and sludge is used to prepare organic fertilizer. Most or all of the treated wastewater is returned to the raw material pre-treatment unit and mixed with cassava to prepare a powder slurry. Some of the remaining wastewater is further treated and discharged after reaching the standard. Excessive ethanol blending ratios can lead to significant reductions in engine power. In current commercial fuel vehicles, the amount of added ethanol is generally no more than 30%. Based on this practical purpose, the amount of ethanol added to the test fuels in this paper does not exceed 30%. This article adds different proportions of cassava bioethanol as a fuel additive to gasoline in an electronic fuel injection engine and mixes the tested mixed fuel by volume. The fuel blends tested in this paper are shown below:

(1) Mixture G100-100% gasoline;
(2) Mixture G90E10-90% gasoline and 10% cassava bioethanol;
(3) Mixture G80E20-80% gasoline and 20% cassava bioethanol;
(4) Mixture G70E30-70% gasoline and 30% cassava bioethanol.

The physical and chemical properties of gasoline-cassava bioethanol fuel blends are shown in Table 2.

### 2.2. Experimental Setup

**Figure 1:**
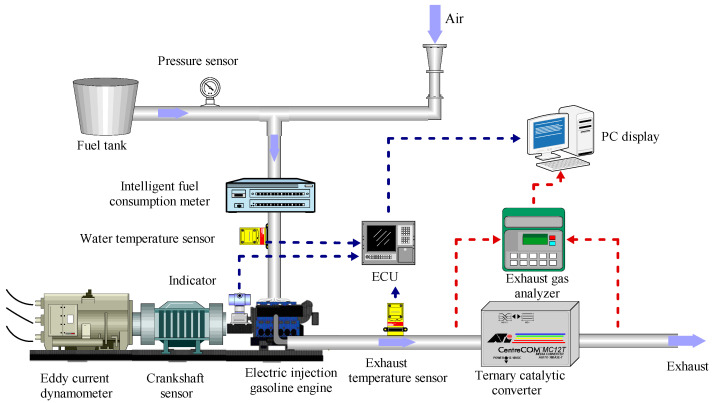
Gasoline engine test system.

The experiments were carried out on an electronic fuel injection gasoline engine. A closed-loop external compressor provided fresh air. To enhance the dependability of the testing, an oil pressure sensor was employed at the outlet, which ensured a consistent oil pressure with an accuracy of ±1% MPa. In addition to the previously mentioned diagnostic equipment, several other specialized tools were employed to ensure accurate performance and precise measurements during the gasoline engine test. The gasoline engine speed and torque were measured with high precision using a dynamometer, with an accuracy of ±1 rpm and ±0.2% F.S., respectively. Emissions of NO_x_, CO, and THC were measured by analyzers with an accuracy of ±5 ppm. Moreover, the gasoline engine was equipped with water temperature sensors and crankshaft position sensors, which were accurate to ±0.5 CA at both ends. The signals from these sensors were sent together with the signals from the waste analyzer to a PC for analysis. The test was conducted at engine speeds of 2000 rpm and 3000 rpm. To further refine the accuracy of the results, all sensors were calibrated prior to the test using industry-standard methods, and the test procedures were carefully controlled and monitored to minimize any extraneous variables that could impact the final results. To ensure consistent heating temperatures in the engine and to avoid any irregularities, the engine was preheated for 6 min before the commencement of the testing process. Figure 1 provides a detailed illustration of the gasoline engine test system used, while Table 3 provides a comprehensive summary of the engine specifications.

### 2.3. Response Surface Method

**Figure 2:**
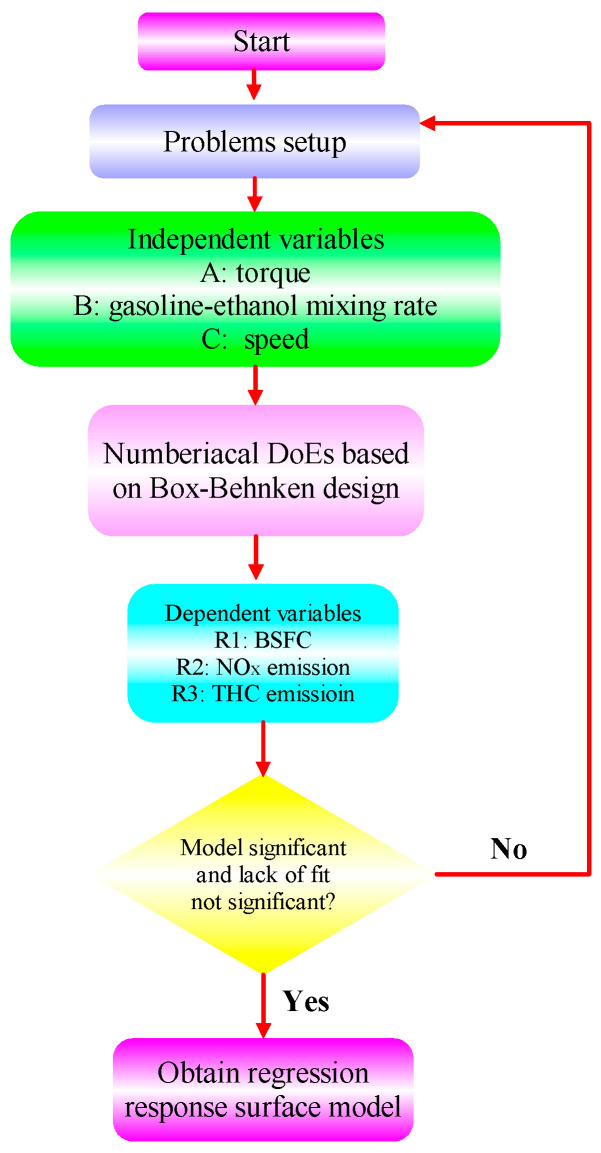
RSM operation process.

RSM is a common optimization algorithm that has been widely used to optimize engine performance and exhaust emissions. Compared to other algorithms, RSM has the following advantages: (1) Efficiency: RSM can explore the optimal solution by establishing a representative mathematical model and optimizing the model. Compared to some traditional optimization algorithms, the RSM usually has higher computational efficiency, especially in situations where the parameter space is complex or the objective function calculation is expensive. (2) Interpretability: RSM establishes a mathematical model to describe the relationship between input variables and objective functions, thus providing an explanation and understanding of optimization results. This is of great significance for understanding the essence of the problem and the impact of variables in the optimization process. (3) Visualization: RSM typically visualizes the impact of input variables on the objective function by constructing a response surface graph. This helps decision-makers better understand the problem and make decisions during the optimization process. (4) Incomplete dependence: the RSM adopts random sampling technology, which can reduce the dependence on actual data to a certain extent. In this section, RSM is used to determine the best solution for different ratios of gasoline-cassava bioethanol fuel blends, speed, and torque regarding performance and emissions [62]. The core of RSM is to bring the input and output parameters into the response model, obtain the mathematical function relationship between the target parameters and the independent variables, and then use a multi-objective optimization method to select the optimal solution according to the desired objective [63,64]. As part of this analysis, engine torque, gasoline-cassava bioethanol mixing rate, and speed were chosen as independent variables, and BSFC, NO_x_, and THC emissions were chosen as dependent variables. The minimum BSFC, minimum NO_x_ emission, and minimum THC emission were used as the objectives for optimization. Figure 2 shows the flow chart of the operation of this experimental RSM.

A total of 17 sets of experiments were run using the RSM approach, including five parallel sets, and the final results are shown in Table 4. 

### 2.4. Uncertainty Analysis and Control Expectations

In order to reduce experimental errors, the article adopts a series of measures. Firstly, in the experimental section, each group of data was subjected to three repeated experiments, and the average value was taken. This helps to reduce the impact of random errors on the experimental results. In addition, five parallel experiments were conducted for the RSM experiment to further reduce the impact of experimental errors. When controlling the expected value of the RSM model, the key input variables that affect the expected value are first determined, such as torque, gasoline-cassava bioethanol mixing rate, and speed. Data collection experiments were then conducted, including 17 random experiments, to obtain a regression model between input variables and output variables. When evaluating the fitting degree of the regression model, it can be observed whether the R^2^ and Adj-R^2^ values in the ANOVA table are close to 1 and whether the difference between Adj-R^2^ and Pred-R^2^ is less than 0.200. These are indicators for evaluating the quality of the model. In addition, in the obtained regression model graph, it is necessary to observe whether the deviation between the predicted value and the actual value is too large. If there is a significant deviation, it may be necessary to conduct a new experiment to determine whether the deviation is caused by experimental errors. If there is still a significant deviation between the expected value and the target value, the combination of input variables can be adjusted, and the optimization steps can be rerun to make the expected value reach a specific target value or fluctuate within a certain range. These measures and steps help to reduce experimental errors, ensure the good quality of the established regression model, and control expected values through optimization steps to achieve optimal performance and results.

## 3. Results and Discussion

Different percentages of gasoline-cassava bioethanol fuel blends were tested for their effects on engine performance and emissions in terms of brake power, torque, BSFC, exhaust temperature, and gasoline engine NO_x_, CO, and THC emissions.

### 3.1. Performance Characteristics

#### 3.1.1. Brake Power

**Figure 3:**
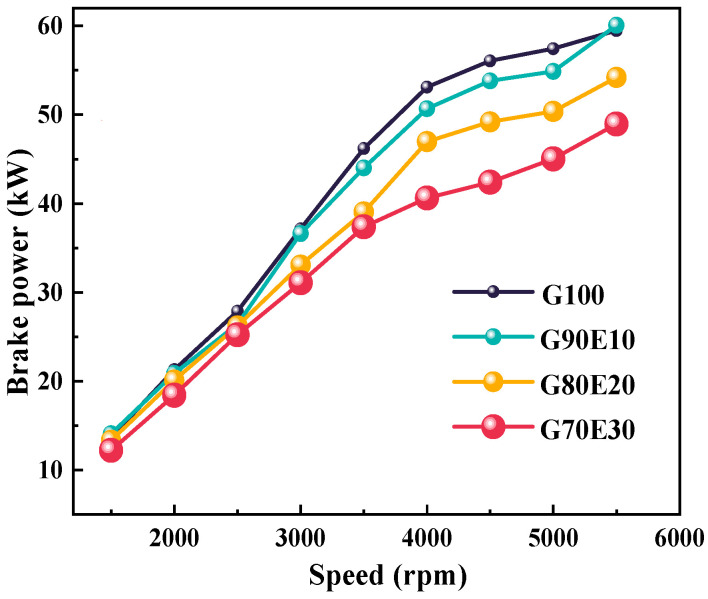
The brake power at various speeds.

At various speeds, Figure 3 illustrates the brake power of gasoline-cassava bioethanol fuel blends in four different ratios. The figure underscores the apparent relationship between the brake power and the blending ratios of the fuel at different engine speeds. As the engine speed increases, so does the brake power of the four different percentages of gasoline-cassava bioethanol fuel blends. All other things being equal, the brake power of the G100 increases more rapidly. At 2000 rpm, with higher ethanol content, the brake power of the mixed fuel was 21.3 kW, 20.8 kW, 20.1 kW, and 18.4 kW, respectively. The low heating value of the fuel blends was 42.9 MJ/ kg, 41.1 MJ/ kg, 39.4 MJ/ kg, and 37.6 MJ/ kg, respectively (see Table 2). As the proportion of ethanol in fuel blends increases, a decline in the lower calorific value can be observed. Consequently, during combustion, the brake power reduces when compared to pure gasoline.

#### 3.1.2. Torque

**Figure 4:**
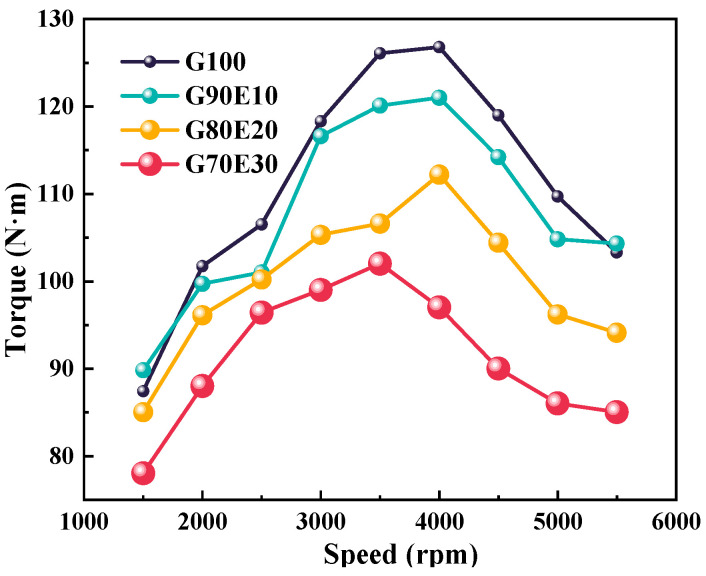
The torque at different speeds.

Figure 4 shows the torque of four proportions of gasoline-cassava bioethanol blends at various speeds. When the speed is accelerated from 2000 rpm to 3000 rpm, the torque shows a tendency to increase. The four different proportions of ethanol gasoline fuel blends all show an inflection point near 4000 rpm when the driving force is at its maximum. In order to reduce noise, engines are often used below the maximum torque, that is, 2000 rpm and 3000 rpm. In this experiment, these are more appropriate choices. At a speed of 2000 rpm, as the ethanol ratio increases, the torque of the mixed fuel is 101 N·m, 99.7 N·m, 96.1 N·m, and 88 N·m, respectively. At a speed of 3000 rpm, the torque was 118 N·m, 116 N·m, 105 N·m, and 99.0 N·m, respectively. Similarly, as the ethanol content increases, the low calorific value and the torque of the fuel blends decrease.

#### 3.1.3. Brake Specific Fuel Consumption

**Figure 5:**
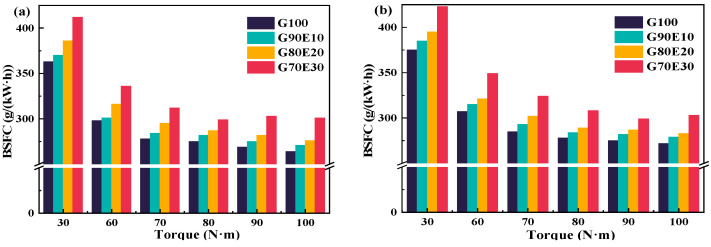
Curves depicting the brake specific fuel consumption of gasoline-cassava bioethanol fuel blends at two different speeds. (**a**) *n* = 2000 rpm; (**b**) *n* = 3000 rpm.

BSFC is an important indicator of fuel economy. The engine consumes the least amount of fuel at moderate speeds and near low speeds. Figure 5 shows the BSFC corresponding to different torques at two different speeds. Under all the conditions tested, the G70E30 always had the highest BSFC relative to the other three fuel blends (under the same operating conditions). The calorific value of ethanol is relatively low, and as the proportion of ethanol increases, the calorific value per unit of energy in the fuel also decreases, resulting in a slight increase in BSFC. This implies, to some extent, that in order to achieve more complete combustion and reduce exhaust emissions, additional fuel consumption needs to be added. In addition, the BSFC of the same fuel is higher at 3000 rpm for the same torque. For example, at a torque of 60.0 N·m, as the ethanol ratio increases, the BSFC of the mixing fuel is 3.02%, 4.60%, 1.60%, and 3.80% higher than those at 2000 rpm, respectively.

#### 3.1.4. Exhaust Gas Temperature

**Figure 6:**
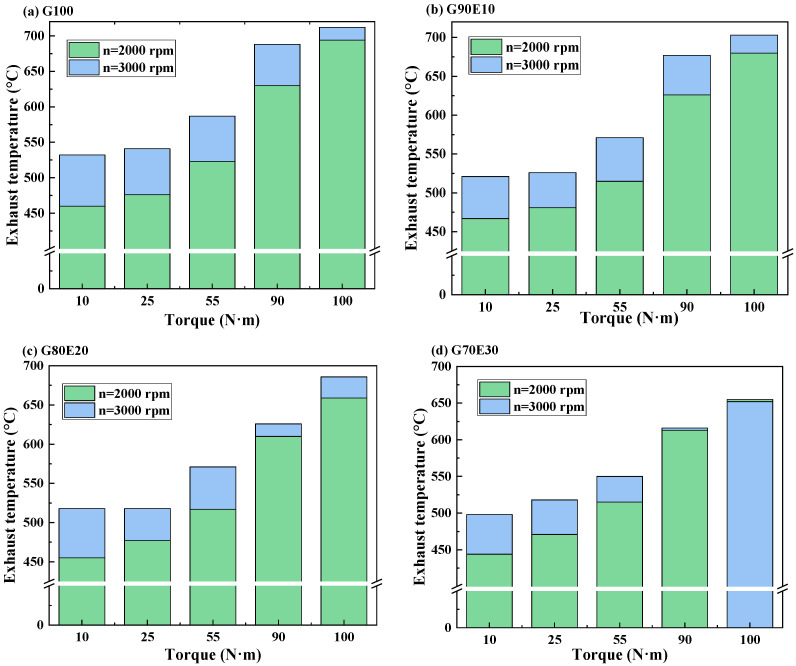
The curves showing exhaust gas temperature variations for gasoline-cassava bioethanol blends with different proportions were analyzed at two different speeds. (**a**) G100; (**b**) G90E10; (**c**) G80E20; and (**d**) G70E30.

The purpose of this section is to examine how the exhaust gas temperature is influenced by the speed and gasoline-cassava bioethanol mixing rate in the fuel blends. In Figure 6, the exhaust gas temperature of gasoline-cassava bioethanol fuel blends with varying ethanol content is presented for two speeds: 2000 rpm and 3000 rpm. Based on the data presented in the figure, it is clear that (1) at the same speed and torque, the percentage of cassava bioethanol added to the fuel mixture increases and the exhaust temperature decreases; (2) when the torque is the same, the exhaust temperature is lower at 2000 rpm. This is because the engine runs at low speeds with relatively low in-cylinder temperatures, resulting in lower combustion and exhaust gas temperatures. Furthermore, as the percentage of cassava bioethanol in the fuel blend increases, it leads to a higher latent heat of vaporization and a lower calorific value. These factors combine to reduce the in-cylinder temperature, resulting in lower combustion and exhaust gas temperatures; and (3) at a speed of 2000 rpm and maximum torque, the exhaust gas temperatures of fuel blends were respectively 2.00%, 5.00%, and 5.60% lower than those of G100 fuel. Similarly, at a speed of 3000 rpm and maximum torque, the exhaust gas temperatures of fuel blends were respectively 1.20%, 3.60%, and 8.40% lower than those of G100 fuel. Badrawada et al. [65] also demonstrated that adding ethanol can reduce the temperature of exhaust emissions. This is because ethanol can increase the oxygen content of the mixed fuel and promote complete combustion.

### 3.2. Emission Characteristics

#### 3.2.1. Variation in Nitrogen Oxide Emission

**Figure 7:**
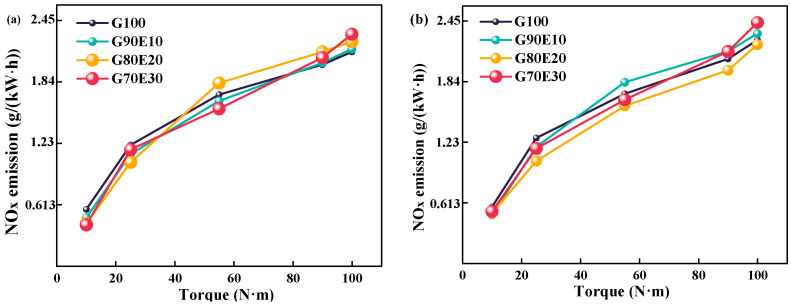
Altering the nitrogen oxide emission profiles observed in fuel blends containing varying proportions of gasoline-cassava bioethanol fuel blends at two different engine speeds. (**a**) *n* = 2000 rpm; (**b**) *n* = 3000 rpm.

This section analyzes the variation of NO_x_ emissions for four fuel blends at different speeds. The generation of NO_x_ is greatly affected by temperature, as indicated by the importance of high-temperature oxygen enrichment and prolonged high-temperature durations for generating NO_x_. The gasoline engine used for the test ran at 2000 rpm with a relatively low exhaust temperature. In addition, the excess air coefficient increased with the increase in cassava bioethanol content in the fuel mixture. As shown in Table 2, the excess air coefficient of fuel blends is 0.920, 0.966, 1.02, and 1.09, respectively. The inclusion of ethanol in fuel blends can improve NO_x_ reduction by bringing the excess air coefficient of the blend closer to 1. At a speed of 2000 rpm, the high latent heat of cassava bioethanol vaporization results in a reduction in in-cylinder temperature. A decrease in this temperature can lead to a decrease in NO_x_ emissions. Figure 7 shows the NO_x_ emissions of different proportions of gasoline-cassava bioethanol fuel blends at the speeds of 2000 rpm and 3000 rpm, respectively. From (a) and (b) of Figure 7, it is evident that (1) NO_x_ emissions change after adding ethanol to gasoline compared to G100. With the increase in torque, NO_x_ emissions increased for all four tested fuels. (2) The NO_x_ emissions of G70E30 start to be higher than those of G100 at 90 N·m of torque at both 2000 rpm and 3000 rpm. (3) Under the conditions of a speed of 2000 rpm and maximum torque, the NO_x_ emissions of fuel blends were found to be 1.30%, 4.70%, and 8.20% higher than those of G100 fuel. Under the conditions of a speed of 3000 rpm and maximum torque, the NO_x_ emissions of fuel blends were found to be 2.80%, −2.00%, and 7.80% higher than those of G100 fuel. The results of this study are similar to those of Luo et al. [66].

#### 3.2.2. Variation in Carbon Monoxide Emission

**Figure 8:**
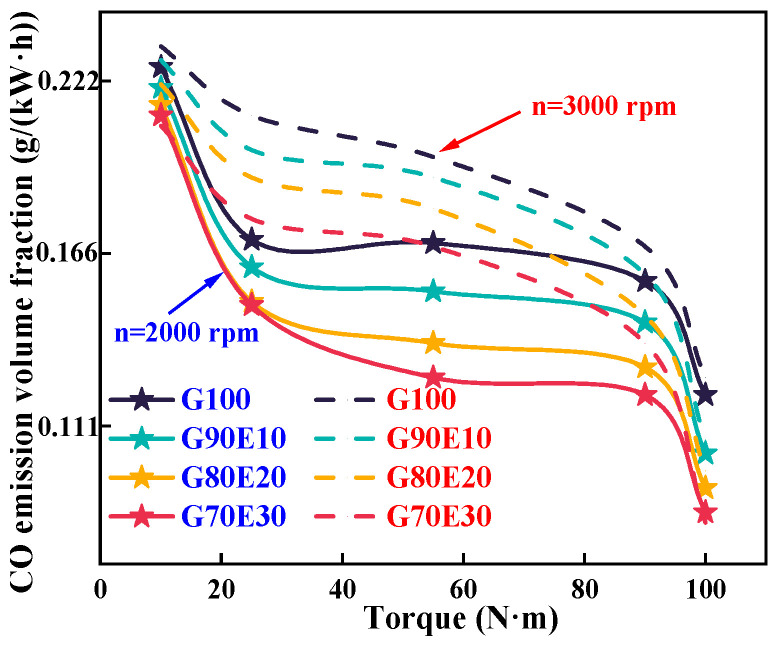
Carbon monoxide emission trends for various gasoline-ethanol fuel blend ratios at two different engine speeds.

Figure 8 shows the CO emissions of gasoline-cassava bioethanol fuel blends at two different speeds. As the speed increases, there is an increase in the volume fraction of CO emissions. This is because higher speeds promote better mixing of gasoline-cassava bioethanol fuel blends, thereby enhancing combustion efficiency and elevating the in-cylinder temperature. So, the eight tested fuel blends increase with increasing speed when other conditions are the same. The density and potential heat of vaporization of the fuel blends increase with the specific gravity of ethanol, but the thermal value of the fuel is reduced. Furthermore, Table 2 illustrates that an increase in ethanol content within fuel blends is accompanied by an increase in the oxygen content of the blends. CO is readily oxidized during a combustion process that is both complete and consistent, so the volume fraction of CO emissions will decrease under all eight conditions. Hence, opting for gasoline-cassava bioethanol fuel blends over pure gasoline fuel can result in a more environmentally friendly combustion process. When the engine was running at a speed of 2000 rpm with maximum torque, the CO emission volume fraction of fuel blends was 15.5%, 22.0%, and 26.0%, respectively, lower than that of G100 fuel. When the engine was running at a speed of 3000 rpm with maximum torque, the CO emission volume fraction of fuel blends was 15.7% and 36.8%, respectively, lower than that of G100 fuel. This research trend is consistent with Sakthivel et al. [67]. In their study, it was found that G70E30 can reduce CO emissions and THC emissions by 75% and 66%, respectively, compared to pure gasoline.

#### 3.2.3. Variation in Total Hydrocarbon Emission

**Figure 9:**
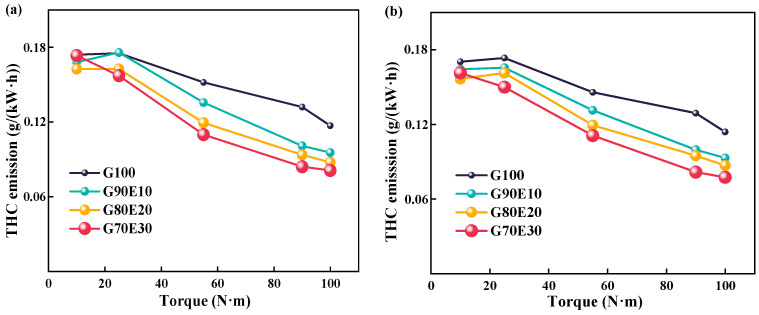
Total hydrocarbon emission trends for various gasoline-ethanol fuel blend ratios at two different engine speeds. (**a**) *n* = 2000 rpm; (**b**) *n* = 3000 rpm.

Figure 9 shows THC emission trends for various gasoline-ethanol fuel blend ratios at two different engine speeds. From this, it can be seen that the higher speed achieves better air-fuel mixing and combustion, which results in less THC emissions. This is in agreement with the experimental results of Yontar et al. [68]. As the torque range was increased from 20.0 N·m to 100 N·m, an inverse correlation was observed between THC emission and cassava bioethanol content in gasoline-cassava bioethanol fuel blends, with a decreasing trend. The reason behind the reduction of THC emissions with an increase in ethanol content in gasoline-cassava bioethanol fuel blends is that the elevated oxygen levels in the fuel (as seen in Table 2) due to the higher cassava bioethanol content lead to a decrease in THC emissions. When the engine was running at a speed of 2000 rpm with maximum torque, the THC emission volume fraction of G90E10, G80E20, and G70E30 was 18.4%, 25.1%, and 30.7%, respectively, lower than that of G100 fuel. When the engine was running at a speed of 3000 rpm with maximum torque, the THC emission volume fraction of fuel blends was 18.4%, 26.3%, and 32.1%, respectively, lower than that of G100 fuel. However, Jin et al. [55] studied the blending ratio of gasoline with E0, E10, E30, E50, and E85 to ethanol-gasoline. They found that the unburned THC emission rate of high-ethanol blended fuel is more than three times higher than that of low-ethanol fuel. This may be due to its increased emissions of alcohol, ethylene, and methane components. Iodice et al. [54] also found similar phenomena in the impact of transient CO and HC emissions during the cold start of ethanol gasoline hybrid fuel. This may be due to the high ethanol blending ratio leading to higher evaporation latent heat under cold start conditions. This leads to a decrease in combustion temperature and speed, leading to a partial combustion process.

## 4. Multi-Objective Optimization

**Figure 10:**
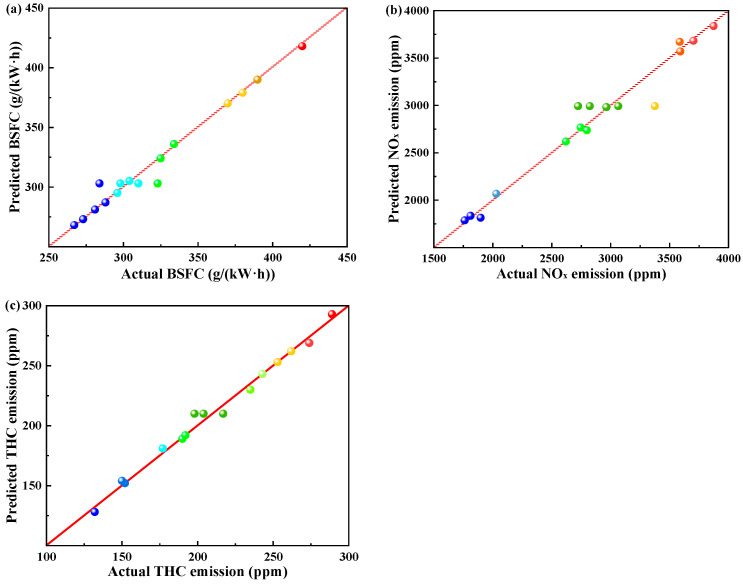
Comparisons of predicted and actual parameters. (**a**) actual vs. predicted values of brake specific fuel consumption; (**b**) actual vs. predicted values of nitrogen oxides; and (**c**) actual vs. predicted values of total hydrocarbons.

In this optimization, gasoline-cassava bioethanol mixing rate, speed, and torque are used as independent variables; BSFC, NO_x_ emission, and THC emission are used as dependent variables. Table 5 shows the dependent variables in the ANOVA table. The comparison between predicted and actual values of dependent variables is depicted in Figure 10, revealing a close similarity between the two sets of values. Meanwhile, the R^2^ and Adj-R^2^ in Table 5 are very close to each other, which indicates that the fit is relatively more accurate and is a superior model. 

### 4.1. Main Factors Affecting Brake Specific Fuel Consumption Emissions

In the experimental process of gasoline engines, the calculated values are not independent due to the influence of combustion and emission boundary conditions. Therefore, this experiment used response surface methodology for optimization design and conducted 17 control experiments. Subsequently, the least squares method was used to fit the response variables BSFC, NO_x_, and THC emission. The response regression equations for BSFC, NO_x_, and THC emissions are Equations (1), (2), and (3), respectively. BSFC can be represented by a second-order regression equation derived from the experimental data, as shown below.

BSFC = 303.66 − 53.754 + 19.88B + 5C − 3.63AB − 1.13AC + 1.12BC + 27.48A^2^ + 7.98B^2^ − 0.52C^2^ (1)

where A is torque (N·m); B is the gasoline-cassava bioethanol mixing rate (%); and C is speed (rpm).

**Figure 11:**
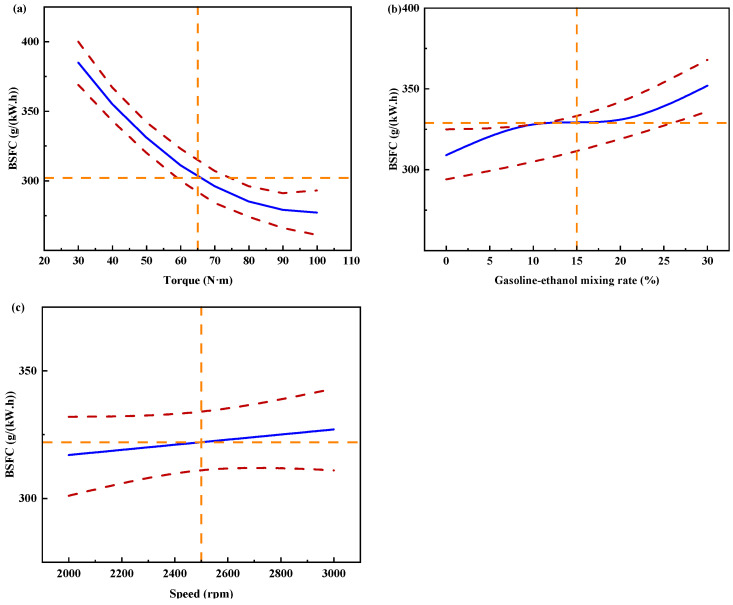
Individual effects on brake specific fuel consumption. (**a**) the independent effect of torque on brake specific fuel consumption; (**b**) the independent effect of gasoline-ethanol mixing rate on brake specific fuel consumption; and (**c**) the independent effect of speed on brake specific fuel consumption.

**Figure 12:**
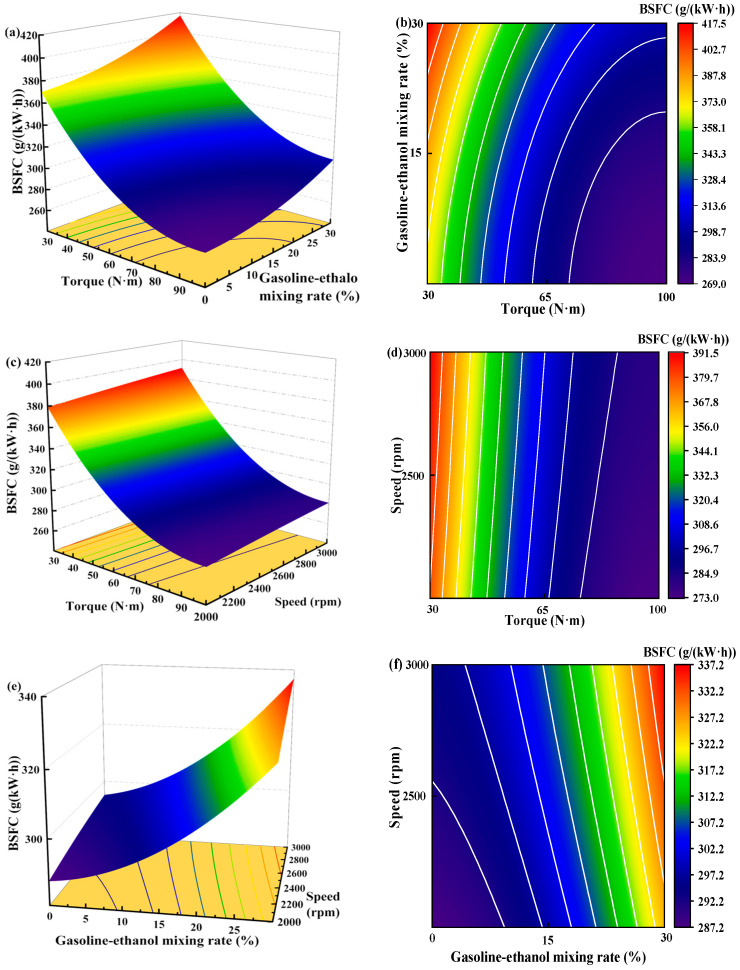
Surface plot of brake specific fuel consumption for different conditions. (**a**,**b**) the joint influence of torque and gasoline-ethanol mixing ratio on brake specific fuel consumption; (**c**,**d**) the joint influence of torque and speed on brake specific fuel consumption; and (**e**,**f**) the joint influence of speed and gasoline-ethanol mixing ratio on brake specific fuel consumption.

Figure 11 shows the individual effects of torque, gasoline-cassava bioethanol mixing rate, and speed on BSFC. As the torque increases, the figure illustrates that the BSFC initially decreases and subsequently rises. The speed has little effect on BSFC. Comparing (a), (b), and (c) of Figure 11, it can be seen that the effect of torque on BSFC is greater than that of gasoline-cassava bioethanol mixing rate and speed, which indicates that the effect of torque on BSFC is the main effect. To further investigate the interaction between variables and responses, the surface plot of BSFC for different conditions was plotted (see Figure 12). Figure analysis reveals that the surface plot color change is notably more conspicuous, indicating a higher level of interaction effect between each pair of factors. A slope analysis of the surface plot reveals that the torque slope is comparatively steep, implying that torque has a more significant impact on the BSFC.

### 4.2. Main Factors Affecting Nitrogen Oxide Emission

NO_x_ emission can be represented by a second-order regression equation derived from the experimental data, as shown below.

NO_x_ emission = 2992.9 + 97.31A − 21.69B + 38.75C + 105.13AB + 15AC + 33BC − 75.14A^2^ − 70.39B^2^ − 201.01C^2^ (2)

where A is torque (N·m); B is the gasoline-cassava bioethanol mixing rate (%); and C is speed (rpm).

**Figure 13:**
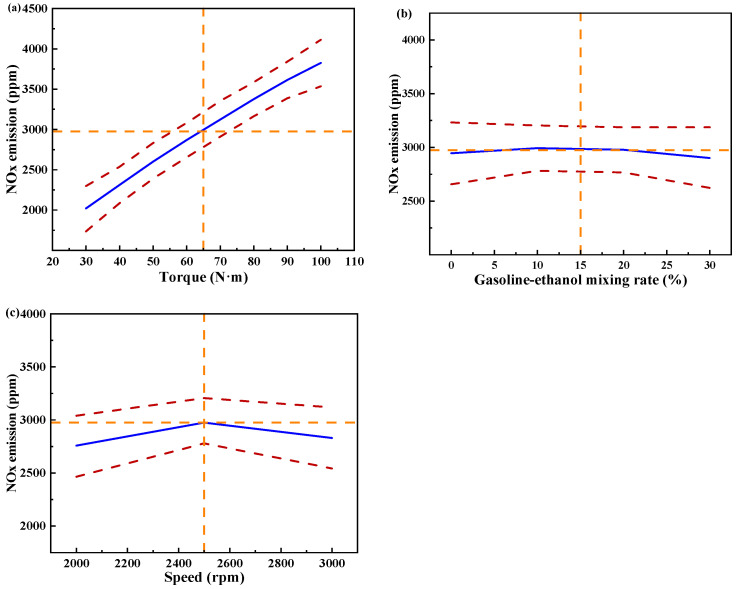
Individual effects on nitrogen oxide emissions. (**a**) the independent effect of torque on nitrogen oxide emissions; (**b**) the independent effect of gasoline-ethanol mixing rate on nitrogen oxide emissions; and (**c**) the independent effect of speed on nitrogen oxide emissions.

**Figure 14:**
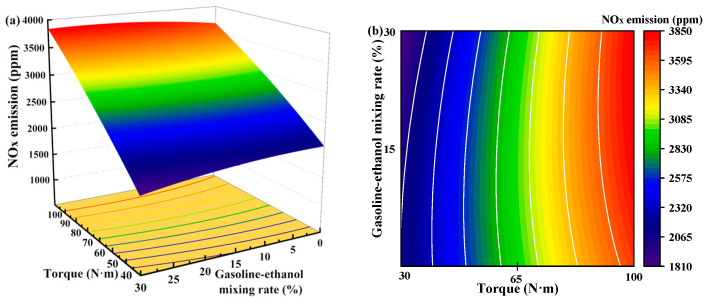

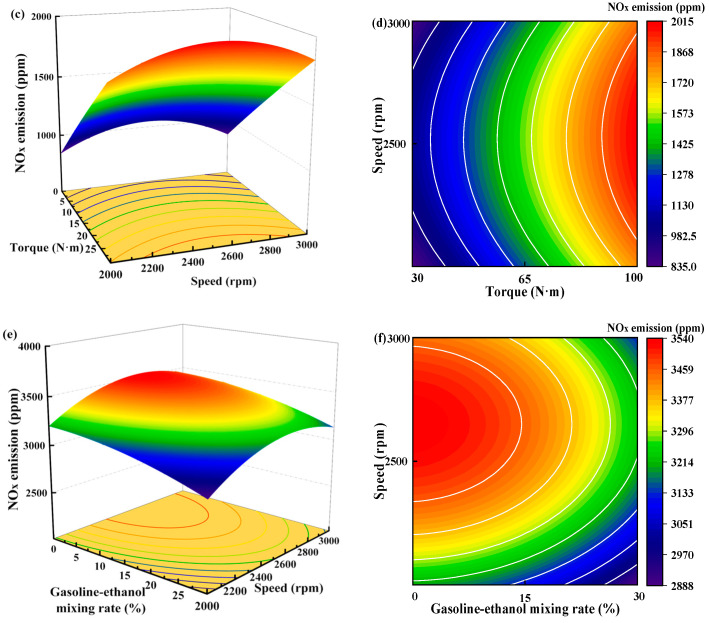
Surface plot of nitrogen oxide emissions for different conditions. (**a**,**b**) the joint influence of torque and gasoline-ethanol mixing ratio on nitrogen oxide emissions; (**c**,**d**) the joint influence of torque and speed on nitrogen oxide emissions; and (**e**,**f**) the joint influence of speed and gasoline-ethanol mixing ratio on nitrogen oxide emissions.

Figure 13 shows the individual effects of torque, gasoline-cassava bioethanol mixing rate, and speed on NO_x_ emissions. The curves of the gasoline-cassava bioethanol mixing rate and speed are relatively flat, as can be seen from the figure. The increase in torque results in a corresponding increase in NO_x_ emissions, suggesting that the main effect on NO_x_ emissions is due to torque. To further investigate the interaction between variables and responses, the surface plot of NO_x_ emission for different conditions was plotted (see Figure 14). The figure clearly demonstrates a more pronounced color change in the surface plot and elliptical contours, which implies a relatively stronger interaction effect between each pair of factors. Analyzing the steepness of the slope in the surface plot, the slope of the torque is relatively steep, which indicates that the torque has more influence on the NO_x_ emission.

### 4.3. Main Factors Affecting Total Hydrocarbon Emissions

Experimental data yield a second-order regression equation that represents THC emission, as follows.

THC emission = 210.3 − 56.63A − 25.88B − 1.13C − 6.25AB + 2.25AC + 5.25BC + 2.6A^2^ + 4.1B^2^ − 3.15C^2^ (3)

where A is torque (N·m); B is the gasoline-cassava bioethanol mixing rate (%); and C is speed (rpm).

**Figure 15:**
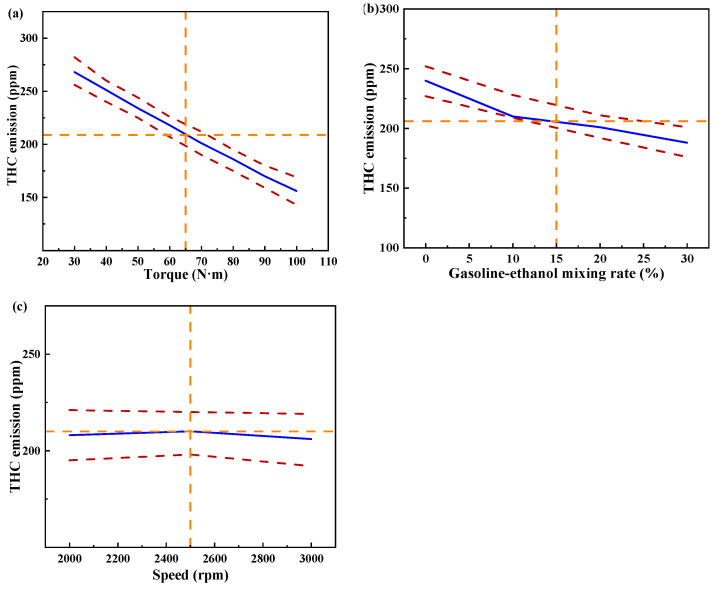
Individual effects on total hydrocarbon emissions. (**a**) the independent effect of torque on total hydrocarbon emissions; (**b**) the independent effect of gasoline-ethanol mixing rate on total hydrocarbon emissions; and (**c**) the independent effect of speed on total hydrocarbon emissions.

**Figure 16:**
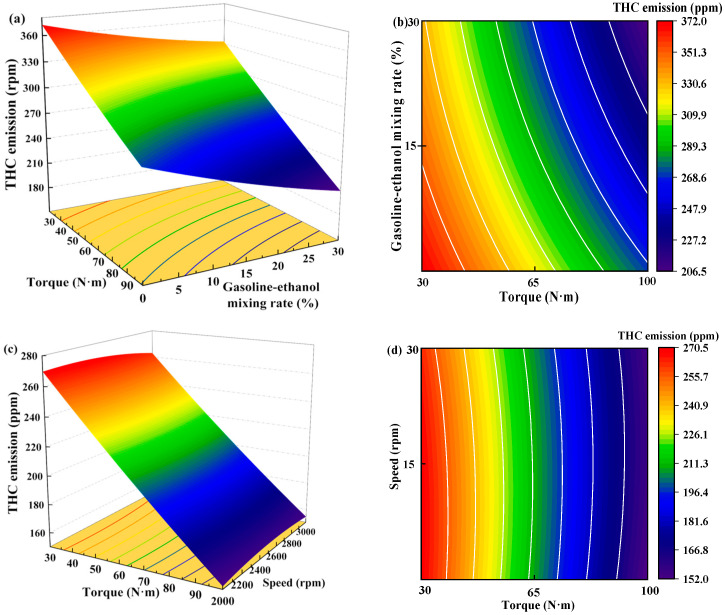

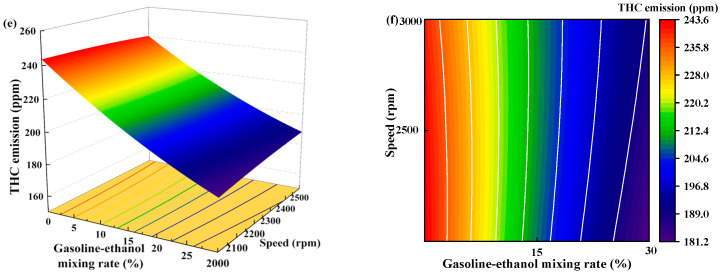
Surface plot of total hydrocarbon emissions for different conditions. (**a**,**b**) the joint influence of torque and gasoline-ethanol mixing ratio on total hydrocarbon emissions; (**c**,**d**) the joint influence of torque and speed on total hydrocarbon emissions; and (**e**,**f**) the joint influence of speed and gasoline-ethanol mixing ratio on total hydrocarbon emissions.

Figure 15 shows the individual effects of torque, gasoline-cassava bioethanol mixing rate, and speed on THC emissions. From the figure, it can be seen that the magnitude of speed has little effect on THC emissions. The effect of torque on THC emissions was greater than the effect of the gasoline-cassava bioethanol mixing rate on THC emissions. In the experimental range, THC emissions decrease with increasing torque and gasoline-cassava bioethanol mixing rates. To further investigate the interaction between variables and responses, the surface plot of THC emission for different conditions was plotted (see Figure 16). The surface plot color change is more obvious, indicating that the interaction effect between the two factors is better. Based on the figure, it is apparent that the slope of torque is steeper, indicating that torque has a stronger impact on THC emissions compared to other factors. THC emission changes more significantly as torque changes from low to high.

### 4.4. Optimum Solution

The Design Expert software finds the requirements that simultaneously meet the experimental objectives (i.e., minimum BSFC, minimum NO_x_ emission, and THC emission). Different optimal solutions are obtained. The optimal solution is considered desirable when its value reaches 0.641. The independent variables are 72.9 N·m torque, 30% gasoline-cassava bioethanol mixing rate (G70E30), and 2000 rpm speed, respectively. The optimal response amounts were 313 g/(kW·h) of BSFC, 2.85 × 10^3^ ppm NO_x_ emissions, and 166 ppm THC emissions, respectively.

## 5. Conclusions and Outlook

In this experiment, the fuel obtained by mixing ethanol and gasoline by the polymer conversion method was used to measure the exhaust emission parameters (including NO_x_, CO, and THC), combustion performance parameters (including BSFC and exhaust temperature), braking power, and torque of this biofuel. With the above experiments and optimization, it was found that the gasoline blend with cassava bioethanol was inferior to G100 in terms of brake power, torque, and BSFC, but better than G100 in terms of CO emissions and THC emissions. As the ethanol content in the gasoline-cassava bioethanol fuel blends rises, a significant reduction in brake power, torque, and exhaust gas temperature occurs. The difference is that the BSFC gets increased to some extent. This is due to the fact that the increased ethanol content increases the oxygen content of the fuel blend mixture. The increase in the calorific value of the fuel causes the BSFC to increase as well. RSM is proven to be optimized for gasoline engines. The best solution derived from this experiment is the independent variable of 72.9 N·m torque, 30% gasoline-cassava bioethanol mixing rate (G70E30), and 2000 rpm speed with a response of 313 g/(kW·h) BSFC, 2.85 × 10^3^ ppm NO_x_ emissions, and 166 ppm THC emissions. The reason for this result is that the oxygen content in the mixed fuel increases with the increase in ethanol proportion, and the octane rating of ethanol is high, which promotes combustion more fully, thus reducing exhaust emissions. In addition, using this ideal hybrid fuel optimization, the exhaust gas temperature is 564 °C and the brake power is 16.0 kW. This optimal solution is of great significance for the optimization of gasoline engines. Applying this optimal solution to practical applications can provide higher fuel economy and lower emission levels. This helps to reduce dependence on oil resources, reduce carbon emissions, and improve air quality. However, this article did not take into account the demand for gasoline-ethanol blended fuel in different regions, models, and uses. For example, certain regions may be more suitable for high ethanol blending ratios, while certain models may be more suitable for low ethanol blending ratios. Future research trends regarding the future of gasoline-ethanol blends include (1) continued research into obtaining different ratios of gasoline-ethanol blends (including different types of engines and uses) to find the best balance of performance and environmental friendliness; (2) future research may explore the integration of ethanol gasoline with electric and hybrid technologies to provide a more sustainable and efficient transportation solution; and (3) ethanol production is also one of the hot topics for future research, including the development of production methods for higher yields and improved disposal of production waste to reduce waste generation.

## Figures and Tables

**Table 1:** The effect of gasoline-ethanol blended fuel on engine emissions.

Ref.	Ethanol Concentration in Blend	Engine Tested	Test Results
[47]	0%, 25%, 50%, 75%, 100%	spark ignition (SI)	NO_x_, CO, HC, CO_2_ ↓
[48]	0%, 50%, 85%	SI	NO_x_, CO, HC ↓
[49]	22%, H100%	SI	NO_x_ ↑ (compared with E22); HC, CO ↓
[50]	0%, 6%, 10%, 15%, 20%	SI	NO_x_ ↓ (except E6); HC ↑, CO ↓
[51]	0%, 5%, 15%	fuel-injected engine	CO, THC ↓, NO_x_-
[52]	0%, 5%, 10%, 15%, 20%	SI	CO ↑ (0–10%), HC ↑, NO_x_ ↓, CO ↓ (10–20%)
[53]	0%, 5%, 10%	SI	CO, HC, CO_2_, NO_x_ ↓
[54]	0%, 10%, 20%, 30%	SI	CO, HC ↓
[55]	0%, 10%, 30%, 50%, 85%	spark ignition direct injection	THC ↑, CO, CO_2_, NO_x_ ↓
[56]	0%, 10%, 20%, 30%	SI	CO ↓ (0–20%), CO ↑ (20–30%), HC, NO_x_ ↓, CO_2_ ↑
[57]	0%, 20%, 50%, 75%, 100%	direct injection engine	NO_x_, CO, HC, CO_2_ ↓
[58]	0%, 2.5%, 5%, 10%, 15%, 20%	SI	NO_x_ ↑, CO, THC ↓
[59]	0%, 10%, 20%, 30%	SI	CO ↓, HC ↑
[60]	0%, 10%, 30%, 50%	SI	CO, HC, CO_2_, NO_x_ ↓
[61]	0%, 5%, 10%, 15%, 20%	SI	CO, HC, NO_x_ ↓

**Table 2:** The physical and chemical properties of gasoline-cassava bioethanol fuel blends.

Fuel Properties	G100	G90E10	G80E20	G70E30
Density (15 °C, kg/m^3^)	745	756	763	787
Kinematic viscosity (40 °C, cSt)	0.480	0.570	0.620	0.690
Lower heating value (MJ/kg)	42.9	41.1	39.4	37.6
Latent heat of evaporation (kJ/kg)	450	491	532	738
Oxygen content (%)	0.000	3.50	6.90	10.4
Theoretical excess air coefficient	0.92	0.966	1.02	1.09
Theoretical air-fuel ratio	14.8	13.5	12.2	10.9

**Table 3:** The primary parameters of the gasoline engine.

Type	Value
Number of cylinders	4
Displacement (L)	1.59
Bore × stroke (mm)	81 × 77.5
Compression rate	10:1
Rated power (kW)	80.0
Rated tongue (N·m)	144
Fuel injection type	Port injection, naturally inspired

**Table 4:** RSM design simulation data.

Torque (N·m)	Gasoline- Cassava Bioethanol Mixing Rate (%)	Speed (rpm)	Brake Specific Fuel Consumption (g/(kW·h))	Nitrogen Oxide Emission (ppm)	Total Hydrocarbon Emission (ppm)
30.0	0.000	2.50 × 10^3^	369	2.03 × 10^3^	289
30.0	15.0	2.00 × 10^3^	378	1.76 × 10^3^	274
30.0	30.0	2.50 × 10^3^	417	1.89 × 10^3^	254
30.0	15.0	3.00 × 10^3^	390	1.81 × 10^3^	262
65.0	15.0	2.50 × 10^3^	303	2.82 × 10^3^	206
65.0	15.0	2.50 × 10^3^	323	2.97 × 10^3^	217
65.0	0.000	3.00 × 10^3^	296	2.81 × 10^3^	235
65.0	30.0	3.00 × 10^3^	336	2.71 × 10^3^	190
65.0	15.0	2.50 × 10^3^	284	2.72 × 10^3^	204
65.0	0.000	2.00 × 10^3^	288	2.79 × 10^3^	243
65.0	15.0	2.50 × 10^3^	298	3.06 × 10^3^	198
65.0	30.0	2.00 × 10^3^	324	2.56 × 10^3^	177
65.0	15.0	2.50 × 10^3^	310	3.37 × 10^3^	226
100	30.0	2.50 × 10^3^	302	3.87 × 10^3^	132
100	15.0	2.00 × 10^3^	273	3.59 × 10^3^	152
100	0.000	2.50 × 10^3^	268	3.58 × 10^3^	192
100	15.0	3.00 × 10^3^	281	3.70 × 10^3^	150

**Table 5:** ANOVA table of dependent variables.

Source	Brake Specific Fuel Consumption Model		Nitrogen Oxide Model		Total Hydrocarbon Model	
	F-Value	*p*-Value	F-Value	*p*-Value	F-Value	*p*-Value
Model	27.9	0.0001	18.68	0.0004	40.8	<0.0001
A	192	<0.0001	160	<0.0001	300	<0.0001
B	26.3	0.001	0.092	0.770	62.6	<0.0001
C	1.67	0.237	0.290	0.605	0.120	0.741
AB	0.440	0.528	1.08	0.333	1.83	0.218
AC	0.042	0.843	0.022	0.886	0.240	0.641
BC	0.042	0.843	0.110	0.753	1.29	0.293
A^2^	26.5	0.001	0.580	0.471	0.330	0.581
B^2^	2.24	0.178	0.510	0.498	0.830	0.393
C^2^	0.009	0.925	4.16	0.080	0.490	0.507
R^2^	0.972		0.960		0.981	
Adj-R^2^	0.938		0.908		0.957	
Pred-R^2^	0.955		0.870		0.925	

## Data Availability

No new data are created.

## Nomenclatures

BSFC	Brake specific fuel consumption
CO	Carbon monoxide
NO_x_	Nitrogen oxides
RSM	Response surface methodology
SI	Spark ignition
THC	Total hydrocarbons
